# Perspective: Treatment for Disease Modification in Chronic Neurodegeneration

**DOI:** 10.3390/cells10040873

**Published:** 2021-04-12

**Authors:** Thomas Müller, Bernhard Klaus Mueller, Peter Riederer

**Affiliations:** 1 Department of Neurology, St. Joseph Hospital Berlin-Weissensee, Gartenstr. 1, 13088 Berlin, Germany; bernhardklausmllr@gmail.com; 2 Center of Mental Health, Department of Psychiatry, Psychosomatics and Psychotherapy, University Hospital Würzburg, Margarete-Höppel-Platz 1, 97080 Würzburg, Germany; peter.riederer@uni-wuerzburg.de; 3 Department of Psychiatry, Southern Denmark University Odense, J.B. Winslows Vey 18, 5000 Odense, Denmark

**Keywords:** neurodegeneration, repulsive guidance molecule A, neuroprotection, repair, oxidative stress, apoptosis, neurogenesis

## Abstract

Symptomatic treatments are available for Parkinson’s disease and Alzheimer’s disease. An unmet need is cure or disease modification. This review discusses possible reasons for negative clinical study outcomes on disease modification following promising positive findings from experimental research. It scrutinizes current research paradigms for disease modification with antibodies against pathological protein enrichment, such as α-synuclein, amyloid or tau, based on post mortem findings. Instead a more uniform regenerative and reparative therapeutic approach for chronic neurodegenerative disease entities is proposed with stimulation of an endogenously existing repair system, which acts independent of specific disease mechanisms. The repulsive guidance molecule A pathway is involved in the regulation of peripheral and central neuronal restoration. Therapeutic antagonism of repulsive guidance molecule A reverses neurodegeneration according to experimental outcomes in numerous disease models in rodents and monkeys. Antibodies against repulsive guidance molecule A exist. First clinical studies in neurological conditions with an acute onset are under way. Future clinical trials with these antibodies should initially focus on well characterized uniform cohorts of patients. The efficiency of repulsive guidance molecule A antagonism and associated stimulation of neurogenesis should be demonstrated with objective assessment tools to counteract dilution of therapeutic effects by subjectivity and heterogeneity of chronic disease entities. Such a research concept will hopefully enhance clinical test strategies and improve the future therapeutic armamentarium for chronic neurodegeneration.

## 1. Introduction

One of the main causes for disability in humans worldwide is onset of neurological disorders, such as stroke and chronic progressive neurodegenerative brain diseases (PND). The most prevalent PNDs are the idiopathic and genetic Parkinson’s disease entity (PD) and the complex of various dementia syndromes, mainly consisting of Alzheimer’s disease (AD), frontotemporal dementia (FTD), mixed dementia (MD), and vascular dementia (VD) [1,2,3]. They are characterized by a common pathophysiologic mechanism, which is aberrant protein aggregation. Well known neuropathological features are β-amyloid and tau-protein enrichment in AD and accumulation of misfolded α-synuclein in PD [4,5]. Incidence of these PNDs will further rise. As an example, estimates of PD prevalence showed a 2.4-fold rise in the last 30 years. Main reasons are an earlier diagnosis associated with better treatment quality and a general rise of human life expectancy [6]. Increased exposure to endogenous and exogenous toxins contributes to a slowly evolving neurodegeneration in the peripheral and central nervous system and accelerates the overall ageing process in a pathological PND related manner. Typical risk factors are pesticides or herbicides, paraquat, rotenone, various metals (i.e., iron, manganese, and lead), gaseous compounds (such as carbon monoxide) and even viruses [7,8]. The rising number of dementia and PD patients will increase the financial burden for health care systems worldwide. To date, it is far from clear, whether the current SARS-CoV-2 outbreak may cause PND like syndromes in the long run, similar to the observed symptomatic PD forms following the 1918 H5N1 influenza pandemic [9]. This review aims to scrutinize current research paradigms for disease modification in PNDs, particularly in PD and dementia syndromes. It scrutinizes current approaches with antibodies against pathological protein enrichment, such as α-synuclein, amyloid, tau, based on post mortem findings. It will finally suggest a more uniform, disease independent therapeutic approach, which aims on neuronal regeneration and repair in the peripheral and central nervous system.

### The Current Situation and Unmet Needs

Considerable research activities in the past 60 years have focused on symptomatic therapies for alleviation of PD. A success story was the introduction of the dopamine substitution concept. It alleviates motor and to a considerable extent associated non motor symptoms in PD since the 1960s [10,11]. At that time Levodopa (L-dopa) was initially applied in an appropriate dose. The introduction of L-dopa therapy was based on findings, that high dopamine levels exist in the basal ganglia and that the dopamine precursor L-dopa counteracts reserpine induced dopamine decrease and associated impaired motor behavior (for review: [12]). The subsequent advancement with the launch of dopamine agonists or inhibitors of L-dopa metabolism reflect a consequent further development of this initial therapeutic principle [13]. These drug alternatives aim to spare oral L-dopa dosing. Reasons for this approach were the still ongoing controversy on L-dopa neurotoxicity due to L-dopa induced oxidative stress, L-dopa related impairment of methylation capacity with consecutive acceleration of ageing processes and the onset of mainly oral L-dopa associated motor complications in PD patients [14,15,16,17]. The future availability of subcutaneous L-dopa pump systems with their more continuous L-dopa brain delivery will noticeably reduce oral L-dopa associated fluctuations of motor and associated non motor behavior in PD. This problem is in the foreground of current clinical PD drug development [18,19]. Similar to PD, non-motor symptoms also gained more and more interest in recent years in dementia. A considerable overlap exists between mechanisms of disease progression between PD and dementia syndromes. Thus, the former focus on the dopamine deficiency in PD, respectively the acetylcholine deficit in AD is superseded by a more widespread view. It also considers the individual different decline of other neurotransmitter systems, like serotonin (5-HT) or norepinephrine [20,21] Generally, particularly AD and PD are related to each other, i.e., by signs of microglial activation and neuroinflammation, and even in terms of neuropathological abnormalities [22,23,24] (Table 1). Similar therapeutic approaches are also employed. As an example, acetylcholine esterase inhibiting compounds and glutamate neurotransmission reducing drugs improve cognitive abilities not only in AD, MD, and VD but also in PD plus dementia syndromes [25,26,27]. 

## 2. Pitfalls of Translational Concepts in Clinical Research

**Figure 1:**
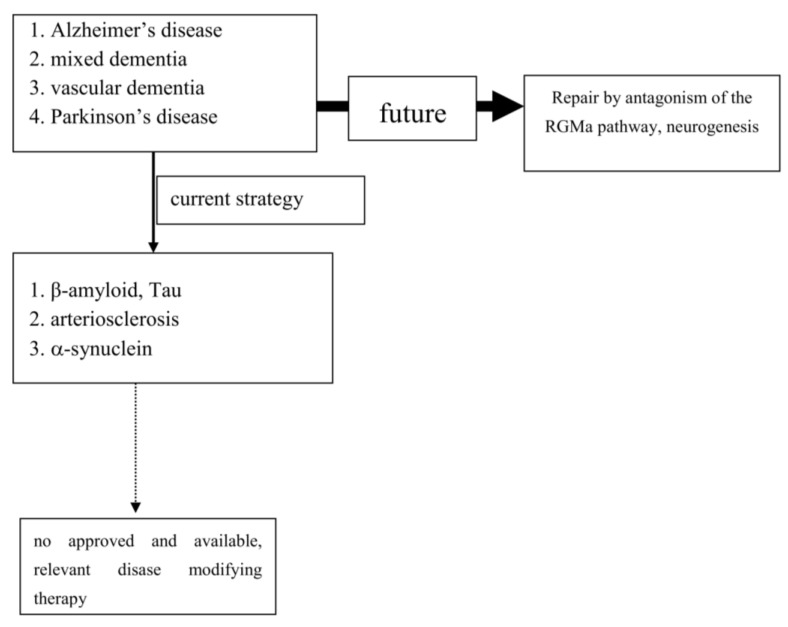
Past and future concepts for disease modification in progressive neurodegenerative brain diseases (PND).

To date, extensive experimental and neuropathological research provided distinct and better insights and understanding of chronic neuronal and associated glial cell death. The predominant responsible and final mechanism cascades are well identified and described in detail [28]. Based on these findings, i.e., antiapoptotic, neuroprotective or oxidative stress reducing compounds, were successfully tested in experimental chronic neurodegenerative and inflammatory disease models [4,29,30,31] (Figure 1). 

However, translation into positive clinical study results has failed so far, as trials on cure or disease modification in PNDs were more or less negative. Even transplantation of neurons or administration of neuronal growth factors was negative (as examples: [32,33]). Stimulation of growth factor synthesis, gene modification, and stem cell applications are still discussed as promising tools [34,35,36,37,38,39,40,41,42]. The unmet need for disease modification, respectively repair regeneration for central nervous system disorders, still exists. No therapy has been approved yet. One reason for these failures may be that chronic neurodegenerative processes result from different heterogeneous, but each other complementing metabolic, pathological cascades (as examples for ground breaking research: [43,44,45] for review: [4,5]). All of them end up in neuronal cell death inducing events, such as apoptosis as the suicide programme of the cell [28,29]. However, the processes, which cause chronic neuronal dying, vary. Moreover neuronal death results in an individually pronounced and variable expression of symptoms in patients. Features of personality, socioeconomic factors, such as education, business conditions etc. may interfere with PND related clinical deficits. The “Neuroplasticity” concept has been suggested to be responsible for the compensation of deleterious metabolic processes and the delayed occurrence of symptoms [46]. These aforementioned heterogeneous components interfere with the value of assessment tools in clinical trials and may dilute potential positive effects of a therapeutic intervention. To demonstrate the benefit of disease modification, validated clinical rating scales were mostly used, sometimes even in combination with functional imaging techniques, i.e., visualization of the dopamine neurotransmission in PD (Table 2). 

Another critical issue is the therapeutic mode of action, which is utilized for disease modification or cure. As an example, antibodies against pathological misfolded proteins were developed based on neuropathological findings. Enrichment of these altered proteins, i.e., in Lewy bodies (LB) or plaques, are looked upon as the main responsible and important, pathological phenomenon in chronic neurodegenerative brain disorders, such as AD or PD [58]. Failures within physiologic activities of protein metabolism may cause protein degradation and misfolding. However, it is far from clear, whether these abnormalities represent a specific process, which is responsible for disease onset and progression [59]. This pathologic protein accumulation may also be the result of an unspecific side reaction of the metabolic cascade during chronic neurodegenerative processes. It may hypothetically only represent well wrapped protein garbage as consequence of physiologic defence mechanisms [59]. The extent of compensatory capacity, the triggering causes and the moment of initiation of these misfolded protein enrichments during the disease process are not known in detail. However, there is consensus that an essential clinical precondition for disease modifying therapeutic concepts is an early diagnosis, when the disease caused damage is low. Accordingly, biomarkers and/or identification of a genetic predisposition may be excellent tools to screen for PND or PND-at risk-individuals. Their availability may theoretically enable a prodromal diagnosis before the onset of motor symptoms (PD) or cognitive decline (AD). To date, PD and AD are mostly diagnosed relatively late in the disease process due to the compensatory “neuroplasticity” phenomenon in the brain. A treatment allocation following earlier prodromal diagnostic screening will also probably reduce the current abundant missing motivation of PND-at-risk individuals for a testing procedure [60]. A positive test outcome without a causal therapeutic approach may cause a heavy burden for further life. Therefore one may scrutinize, whether the subsequently described clinical research pattern for disease modification is appropriate in chronic neurodegeneration, such as dementia or PD.

## 3. Dementia Syndromes

Post mortem neuropathological brain investigations describe an accumulation of plaques and tangles with β-amyloid- and/or tau protein pathology in AD. Dystrophic neuritis, astrogliosis, neuropil threads, and microglial activation with neuroinflammation has also been reported [22,23,24,61]. These changes result in an acetylcholine deficit, which is mainly looked upon as responsible for the cognitive impairment. Morphological and functional imaging techniques, such as magnetic resonance imaging tomography or positron emission tomography (PET) with various radiotracers, were developed for visualization of brain function and neuroinflammation [49,62]. Particularly, PET shall help to interrogate the biological mechanisms of disease initiation, progression, and assessment of successful, potential future disease modifying therapies. It also serves as a diagnostic tool. In AD trials, PET is used for a better patient cohort characterization. The enrichment of the radiotracer [^18^F]-AV-45 is employed for the determination of β-amyloid plaque density [63,64,65]. This approach visualizes “pure” AD forms, particularly in combination with screening for genetic risk factors. The most known one is the *ApoE* polymorphism. There are three major isoforms, ApoE2, ApoE3, and ApoE4. They impact the risks for developing AD. Carriers of the homozygous *ApoE4* allele have the highest AD risk, *ApoE3* is considered as normal and *ApoE2* is looked upon as protective [66,67]. Accordingly, preclinical researchers developed genetically predisposed AD models. Novel AD drugs were screened and tested in these uniform AD models, designed on the basis of a specific genetic predisposition or acetylcholine deficiency. As an example, antibodies against β-amyloid aggregation or tau pathologies were first successfully investigated in AD animals [68,69]. These experimental investigations proved their efficacy. However compounds failed in clinical trials (for review: [68,69]). This is no surprise for clinicians involved in the real world maintenance of dementia patients. The variability of symptoms, observed in the clinical maintenance of AD patients, was not considered. The most common dementia syndrome represents a mixture between vascular dementia and AD on top of additional manifestations of further comorbidities, i.e., diabetes, hypertension, metabolic syndrome, etc. All of them predispose for dementia and acceleration of ageing. Out of this spectrum of dementia forms, AD is looked upon as the most popular form of dementia. AD allows a specific therapeutic approach based on one pathophysiologic mechanism [5,27]. The strict inclusion criteria of clinical trials mostly try to select “pure” AD patients with nearly no concomitant disorder. However, even this study population represents a broader spectrum of clinical symptoms with individual differing rate of progression. Efficacy of tested compounds is mainly determined with repeat performance of standardised neuropsychological cognitive batteries and clinical rating scales. Both of them were suitable to demonstrate symptomatic effects in trials on the cognition enhancing effects of acetylcholinesterase inhibitors [25,26,68]. However, performance training, which is often executed during dementia trials by caregivers and patients, may dilute their value. As example, the Mini Mental State Examination is often used as selection criterion in dementia trials, but its scores have a considerable bias by the educational level of the patient [70,71,72,73]. Instruments, like the neuropsychiatric inventory, lack of assessment of psychopathological phenomena in relation to exogenous influencing factors, i.e., well-being of caregivers, etc. Does this laboratory study condition within a clinical trial reflect real life of health care in AD? Clinicians point out that non cognition related signs, i.e., motor signs, are further essential quality of life limiting factors and influence progression [74]. To date, the suitability of these assessments for verification of disease modification was scrutinized only to a certain extent. However, use of these clinical rating scales may be responsible for the failed translation of therapeutic interventions on tau- and β-amyloid metabolism from successful preclinical experiments into positive clinical studies in AD. These considerations may be additional reasons, why the Phase III study programs with the two anti-Aβ monoclonal antibodies, bapineuzumab, and solanezumab, were negative [25,26,68]. The discussion on a possible approval of aducanumab, which showed mild effects in faster progressing AD patients, is still ongoing. In contrast to these aforementioned approaches in diagnosed patients, another promising preventive concept is vaccination. Aggregated β_1-42_-amyloid was employed in animal models for active immunization. Clinical vaccination trials were stopped due to onset of meningoencephalitis in up to 6% of the participants [75,76,77,78]. As a next step, a shift from active to passive immunization is now under way in AD models [75]. It is regarded as more safe, to be better controlled and may also be efficacious [79,80,81,82]. However, will vaccination against aggregated β-amyloid plaques be beneficial for other pathological processes associated with AD, like the tau-protein pathology? 

### Pragmatism of Clincians

Currently the only way to counteract AD onset or to modify the course of AD is prevention of the major AD risk factors. Arteriosclerosis disease, type II diabetes, midlife hypertension, midlife obesity, metabolic syndrome, smoking, and physical inactivity are looked upon the most common ones. Reduction of these life style associated predisposing risk factors with preventive, more educational approaches is suggested for dementia-at risk-individuals [83,84]. 

## 4. Parkinson’s Disease

Currently it seems that these misconceptions of AD research will be repeated in PD. PD is mostly diagnosed, when approximately 60% of dopaminergic axons and 30% of nigral dopaminergic neurons are already gone. At that stage motor symptoms appear [85]. Accordingly one may scrutinize, whether testing of a disease modifying therapy for the remaining 70% dopamine synthesizing neurons in the most affected nigrostriatal area in PD may provide clinical relevant changes for the future disease course at all. The still surviving neurons have already lost most of their axons. Therefore they are primed for cell death. Better awareness for non-motor signs and initiatives for earlier detection of PD were initiated. They shall characterize the so-called “prodromal” or “premotor” interval. Heterogeneity of symptoms and the individually differing progression complicates the inauguration of a clinical, reliable classification concept of early PD signs. Moreover various subtypes of the disease entity PD exist [86,87]. No validated, reliable, specific, easy to apply biomarker for an earlier detection of PD or a PD screening was found to date. Still, the LB accumulation with misfolded α-synuclein protein aggregation is looked upon as the most important neuropathologic PD hallmark. Related syndromes, such as Parkinson’ disease dementia (PDD) and dementia with Lewy bodies (DLB), also show these neuronal inclusions. A widely cited model of the dopaminergic neuron degeneration, the Braak model, even considers PD as α-synucleinopathy and sets LB pathology as the essential lime light [88,89,90]. This process of slow neuronal dying follows a certain pattern, for instance starting in the gastrointestinal tract, rising over the brainstem with further spreading all over the brain. This hypothesis is matter of controversy [90,91,92]. No correlations between LB pathology and loss of dopamine generating cells or clinical features of PD have been found yet. No correlations between Braak staging and dopaminergic neuron density in the substantia nigra appeared [93]. These negative results may hypothetically suggest that LB pathology is not essential for nigral degeneration in PD. Post mortem investigations in Leucine-rich repeat kinase 2 (LRRK2)- and Parkin-related PD patients or in PD models with 1-methyl-4-phenyl-1,2,3,6-tetrahydropyridine (MPTP) induced nigrostriatal neuronal loss and dopamine deficit show no LB occurrence [94]. Currently employed PD models mainly focus on the loss of dopamine synthesizing nigral neurons. However, chronic neuronal dying also takes place in other neurotransmitter systems in PD, both in the periphery and in the brain [91]. Various neuronal cell types degenerate in an individually different and pronounced manner in various brain regions. This is mirrored by a heterogeneous compilation of motor and non-motor symptoms in PD patients [95,96,97]. Employed in vitro and in vivo PD models mimic the chronic progressive, slow sometimes relapse like progression of neuronal dying to a limited extent. Toxin models with, e.g., 6-OH-dopamine, rotenone, or MPTP application, induce nigrostriatal dopamine deficiency [98]. They only reflect the motor impairment in PD and often result from an acute event after a singular toxin administration. Accordingly, these models mirror the various therapeutic effects of dopamine substitution even in combination with PET, which employs radiotracers for the nigrostriatal dopamine neurotransmission. These functional imaging techniques are also employed in the clinical routine, i.e., as instrument for the confirmation of diagnosis. They are able to mirror PD progression to a certain extent. Effective disease modification was shown in long term studies with PD patients. They showed a lower advance rate with dopamine agonist treatment alone compared with L-dopa/dopadecarboxylase inhibitor (DDC-I) administration as monotherapy (as an example: [99]). This technique was additionally employed for the comparison of disease progression during treatment with two L-dopa/DDC-I dosages against placebo. Progression was faster in the 600 mg L-dopa/carbidopa arm after nine months compared with 300 mg and placebo [100]. No difference appeared, when the effects of dopamine agonist monotherapy versus placebo treatment were investigated in a clinical long term trial with a delayed start design with the same imaging technique as tool for determination of PD progression [48]. The combination of the employed assessment techniques in these aforementioned trials showed that performance of disease modification is possible in chronic neurodegeneration. Avoidance of L-dopa, respectively low L-dopa dosing, is beneficial in the long run in PD, which is still matter of debate [14,15,16,17]. Clinical evidence for a disease-modification/neuroprotective action was also investigated with the MAO-B inhibitors selegiline and rasagiline with various clinical defined endpoints only without the use of expensive functional imaging techniques. The subsequent description of these clinical trials will mirror the complexity of these trials and the interpretation of their outcomes. 

### 4.1. Excurs: Clinical Research on Disease Modification in PD with MAO-B Inhibition

DATATOP (Deprenyl and tocopherol antioxidative therapy of parkinsonism) was a prospective, randomized, double-blind, placebo-controlled trial with 800 PD patients. Treatment naïve PD patients were randomised to either selegiline or α-tocopherol (vitamin E), a combination of both or placebo. Then they were followed until clinical deterioration asked to initiate an additional symptomatic L-dopa therapy. This was interpreted as primary endpoint. Selegiline, but not α-tocopherol, delayed L-dopa treatment compared with placebo after 15 months. The trial was stopped, when 24.3% of participants taking selegiline compared with 43.9% of those without selegiline reached the primary endpoint. Then a change of protocol was performed. Therapy was withdrawn from 367 participants, who did not meet the primary endpoint. These PD patients were evaluated again after one and two months. From baseline to the end of the washout, patients under selegiline therapy showed a slower disease progression according to their Unified Parkinson’s Disease Rating Scale (UPDRS) outcomes, compared with those without selegiline. In comparison to patients without selegiline therapy, the ones on selegiline were slightly better during the washout period in UPDRS part III (motor examination). In the subgroup of DATATOP patients who were not on L-dopa yet (n = 310), no benefit of early initiation of selegiline therapy was found. Those patients with selegiline therapy since trial initiation reached the endpoint of necessary L-dopa therapy sooner than those who had switched from placebo to selegiline. A total of 368 of the DATATOP participants on the L-dopa/carbidopa plus selegiline regimen were further randomised to continue with selegiline or to switch to placebo. The patients on selegiline deteriorated slower compared with those who were randomized to placebo. These results only suggest a beneficial effect of selegiline on disease progression once the patients were on supplemental L-dopa therapy. No beneficial effect was found with vitamin E therapy. The main limitation of this study was a potential confounding symptomatic effect of selegiline on outcomes in combination with the observed placebo effects [56]. SIN-DEP-PAR (Sinemet-Deprenyl-Parlodel) was a prospective, randomized, double-blind study. It randomized 101 patients with mild to moderate PD to either selegiline (10 mg/day) or placebo. Three days later either L-dopa/carbidopa or bromocriptine was supplemented and dosed based on clinical response. Patients on bromocriptine were allowed to add L-dopa/carbidopa if necessary after an escalation of bromocriptine to 20 mg/day or the maximally tolerated dose. Both cohorts were matched and remained in this study for 12 months. Then, a two-month lasting washout period followed. L-dopa and bromocriptine were stopped one week before the final scoring at month 14. At the final evaluation, selegiline treated patients showed less worsened disability compared with the group, which was not on selegiline (0.4 versus 5.8 points on total UPDRS score; *p* < 0.001). This effect was particularly observed in L-dopa/carbidopa treated patients. Patients on selegiline (*p* < 0.01) deteriorated less (total UPDRS score: −1.7) compared with those on placebo (+4.8). A total of 23 patients were again rated 2 weeks after stop of L-dopa/carbidopa and bromocriptine again. Selegiline treated patients had less change in their UPDRS scores from baseline compared the ones on placebo [57]. The SELEDO (from selegiline plus L-dopa) trial was a prospective, double-blind study over an interval of five years, which investigated the efficacy of selegiline addition to L-dopa. An optimum titration of L-dopa to individual requirements was done at study start. Then the 116 PD patients were put on either selegiline or placebo. The subsequent necessary L-dopa dose escalation depended on the clinical features. The primary endpoint was defined as the moment when the needed L-dopa dose to control symptoms was 150% or more of the initial dose. Median time to reach this endpoint was 4.9 years in the selegiline group in comparison with 2.6 years in the controls. After five years of treatment, only 50.4% of the selegiline treated patients reached this endpoint in comparison with 74.1% of the placebo treated participants. Both outcomes were significant [101]. These aforementioned selegiline long term trials showed that combination of selegiline and L-dopa provides a greater clinical benefit and less progression than L-dopa monotherapy, when the need for L-dopa is looked upon as an indirect clinical biomarker for disease severity. A certain beneficial effect on the course of the disease was shown with the 1 mg dose in the ADAGIO trial but not with the 2 mg daily dose of rasagiline employing a delayed start design. There are a lot of hypotheses on the failure of the 2 mg arm, which is not explained to date. This controversial outcome only occurred in the ADAGIO (A Randomized Placebo Controlled Study to Show That Rasagiline May Slow Disease Progression for Parkinson’s Disease)—but not in the earlier performed TEMPO (A controlled trial of rasagiline in early Parkinson disease) study. TEMPO showed beneficial effects of rasagiline on disease progression. However, the baseline UPDRS values and durations of disease were lower in the ADAGIO-((UPDRS): 20.4(8.5) (median(SD)); (duration of disease): 4.5(4.6) (months)) than in the TEMPO-participants (25.0(10.84); 12.1(13.2)) [51,52,102]. To date, a certain disease modifying effect of these MAO-B inhibitors is still under discussion and has not been approved as disease modifying effect (Table 2). Duration of trials, sensitivity of applied rating instruments and inconsistency of outcomes are important arguments.

### 4.2. Current Ongoing Clinical Research Strategies on Disease Modification in PD

These aforementioned studies included patients with sporadic PD [58]. Thus, environmental epigenetic influences, chronic toxin exposure or other still unknown for instance viral or bacterial infections may be main hypothetical causes for PD onset. In the past, these sporadic PD patient cohorts were also employed for performance of transplantation trials with fetal dopamine synthesizing neurons or inhibition of progression with possible disease course modifying therapies (as examples: [33,40]). They were negative, similar to the aforementioned neuroprotection trials in PD patients with selegiline, rasagiline and free radical scavengers, like coenzyme Q10 or tocopherol [47,52,56,103] (Table 2). Currently new attempts are under way with less heterogeneous patient cohorts based on genetic vulnerability factors for PD. Generally, there is also no doubt, that a certain genetic impact is present in some forms of PD. Experimental research demonstrated the corresponding pathological pathways following the initial description of α-synuclein mutations. More than 20 so-called “PD genes” with a different extent of penetration are known nowadays and are allocated to be responsible for sporadic PD. Genetic alterations and mutations in familial PD forms, such as SNCA, Parkin, LRRK2, DJ-1, PINK-1, and UCHL-1, only account for approximately 10% of idiopathic PD patients. Age of onset and clinical symptoms are variable, as example convincingly demonstrated in Glucocerebrosidase (GBA) mutation carriers [104,105,106,107,108]. Neuroprotection trials are again under way in these GBA mutation carrying PD patients. Currently there are also new research initiatives under way in PD with antibodies against α-synuclein based on the corresponding neuropathological findings of protein misfolding in Lewy bodies [109,110]. Various drug mechanisms aim to reduce misfolded α-synuclein and thus disease progression. They focus on boosting of autophagic/lysosomal clearance, reduction of α-synuclein mRNA by modulating histone deacetylase or RNA interference with decreased expression of α-synuclein [58]. Further therapeutic concepts focus on the impeding of the α-synuclein multimerization with heat shock proteins, dissociation of existing misfolded α-synuclein aggregates with small molecules, blocking of α-synuclein entry through receptor blocking, prevention of α-synuclein transport from cell to cell and immunotherapy with neutralization of α-synuclein extracellularly or intracellularly with nanobodies [111]. There is one but essential disadvantage. The basis for this therapeutic concept is always a more or less singular molecular pathology drug approach based on a neuropathological post mortem finding. Can one really assume that this will be sufficient to slow down progression of sporadic PD? Its onset and advance are characterized by various triggers [95]. They have a multifactorial, possibly multigenetic origin. Further different pathological factors for disease progression exist. All of them are responsible for a heterogeneous compilation of clinical symptoms and their progression. Similar to AD, vaccination as preventive approach is also discussed, but outcomes of a vaccination trial in PD are not reported yet.

## 5. Conclusions

Cure or modification of progression in dementia, particularly AD and PD, is an important unmet need. Clinical trials, which aimed to translate promising experimental research outcomes into relevant positive results, were negative. No therapy has yet been approved despite well identified final main neuronal and related glial cell death mechanisms mostly on the cellular level. Multifactorial origins, heterogeneity of clinical symptoms, variability of each other complementing disease mechanisms and progression are the mostly likely reasons.

## 6. Outlook

Experimental research convincingly described a number of cellular pathways leading to chronic neuronal degeneration and death in PNDs. Examples are mitochondriopathy, dysfunction of the ubiquitin/proteasome system, oxidative and nitrosative stress, dysregulation of heat shock response, altered iron metabolism and vesicular transport systems, apoptosis, necrosis, autophagy, microglial activation combined with neuroinflammation [23,62,112]. Therapeutic concepts were, and are, developed based on findings, such as that the pro-inflammatory TNF-alpha cytokine is able to modify neuronal plasticity, maturation, and function of human cholinergic neurons also by epigenetic mechanisms [61]. All of these disease related alterations and their possible therapies will consequently change neurotransmission pathways [113]. However to date, preventive or PND progression delaying therapeutic strategies failed following translation into clinical study programs (as an example: [47]). Even the current clinical testing of specific antibodies against certain proteins, which accumulate in the various sporadic PNDs subtypes, may probably fail as shown in AD. This may suggest that the enrichment of these proteins is not specific. Their accumulation overlaps between various clinical PND pictures. This protein enrichment in LB may hypothetically only represent a defence mechanisms against the disease process itself, but do not cause it (Table 1; [114]). To date all clinical studies, which aimed to demonstrate neuroprotection or disease modification, i.e., in PD and AD, showed that research on a specific pathological disease mechanism does not lead to an essential therapeutic innovation in terms of disease course modification (Figure 1). Therefore, the underlying research method is worth to be considered for a modification. As an alternative to this misconception, one may consider the stimulation of an existing repair system in the peripheral and central nervous system as a more promising research paradigm [115,116,117,118,119,120]. Therapeutic strategies, which antagonize the repulsive guidance molecule A (RGMa) pathway, are worth for further development in clinical trials. A RGMa increase in the substantia nigra was found by in situ hybridization and immunohistochemistry in neuromelanin-positive neurons in post-mortem tissue of treated PD patients [116]. It may also be related to L-dopa administration and associated oxidative stress generation to a certain extent [44,121]. Extracellular RGMa inhibits axon regeneration and therefore may accelerate demise of neurons [122,123,124]. However, targeting the RGMa pathway with antibodies or neutralization, respectively, antagonism of the neogenin receptor activity, may start regeneration not only in acute, but also in chronic inflammatory and neurodegenerative disorders [46,125,126,127,128] (Figure 1). It is well known, that considerable metabolic similarities exist both in the peripheral and central nervous system. Therefore, it is hypothesized that other syndromes than PD and AD, may also respond to this approach [118,120,123,129,130]. It may restore neuronal function in the long term as a general concept for repair and may weaken efficiency of toxin exposure [46,116,117,125,126,131,132,133]. Well designed, clinical long term trials with RGMa antagonizing approaches are urgently needed in multiple sclerosis, PD, dementia syndromes, stroke, or spinal cord injury. Neuropathies (NP), diabetic retinopathy, Guillian Barre syndrome and amyotrophic lateral sclerosis are particularly suitable disorders. They allow testing of this approach in rather homogenous, well defined study cohorts with objective assessment tools, such as visual function and visual evoked potentials in retinopathy, or sensory or motor nerve conduction assessment in NP. RGMa antagonism may probably also help to counteract heterogeneous neurological deficits as consequence from severe viral infections, including SARS-CoV-2. Currently two different neutralizing RGMa antibodies (ABT-555; MT-3921) are in phase 2 clinical trials in spinal cord injury. In addition ABT-555 is in phase 2 clinical trials in progressive and relapse-remitting multiple sclerosis and in ischemic stroke. A positive outcome of these clinical trials will support this strategy for regeneration and repair in the damaged human nervous system. A further important pathological mechanism-of-action in PNDs is the potential inhibition of neurogenesis by the RGMa-neogenin pathway also in PD and dementia syndromes [115,118,119,120,134]. Neurogenesis also occurs in the adult human brain, i.e., in the dentate gyrus or the subventricular zone. RGMa blocks neurogenesis in these areas [123,135]. As shown in the hippocampal dentate gyrus, blocking of RGMa promoted formation of new neurons [115]. Targeting RGMa by antibodies may promote neurogenesis in the adult human brain of PND patients. An increased neurogenesis may also improve motor symptoms in PD or cognitive deficits in dementia. This is an alternative to cell replacement and stem cell concepts. Both have a focus on specific cell types only in contrast to the potential of RGMa antagonism in chronic neurodegeneration [34,35,49,136,137,138,139].

## Figures and Tables

**Table 1:** Interactions of various pathological proteins and disease.

PD	Lewy Bodies (α-synuclein)
DLB	Lewy bodies plus β-amyloid
DLB + AD	Lewy bodies plus β-amyloid plus neurofibrillary tangles
PDD	Lewy bodies plus β-amyloid plus neurofibrillary tangles (tau-protein)
mixed variants (PSP + LB)	Lewy bodies plus neurofibrillary tangles
neurofibrillary tangles dementia (PSP, CBD)	neurofibrillary tangles
AD	β-amyloid plus neurofibrillary tangles
pathological ageing	β-amyloid

AD, Alzheimer’s disease; CBD, corticobasal degeneration; LB, Lewy bodies; PD, Parkinson’s disease; PDD, Parkinson’s disease dementia (modified from 114).

**Table 2:** Important trials in Parkinson’s disease, which aimed on modification of progression.

	FIT	Scale	Comparison	Result	
LEAP-Study	No	MDS-UPDRS	Early vs. later application of L-dopa	faster PD progression with longer L-dopa exposure	[17]
Coenzyme Q 10	No	UPDRS	Coenzyme Q 10 vs. placebo	Negative	[47]
PROUD-Study	Yes	UPDRS	delayed start design; Pramipexole vs. Placebo	no difference	[48]
REAL-PET	Yes	UPDRS	Ropinirole vs. levodopa	positive in favor of ropinirole in terms of PET outcomes	[49]
PELMOPET	Yes	UPDRS	Pergolide vs. Levodopa	no difference due to use of different PET machines for intraindividual comparisons	[50]
ADAGIO	No	UPDRS	Delayed start design Rasagiline 1 mg or 2 mg vs. placebo	positive for 1 mg, but not 2 mg	[51,52]
TEMPO	No	UPDRS	Rasagiline	Positive effect of rasagiline	[51,53]
Pramipexole vs. levodopa as initial treatment for Parkinson disease: double blind trial.	Yes	UPDRS	Pramipexole vs. Levodopa	Tendency in favor of pramipexole (Pramipexole: 20.0% (14.2%) vs. LD: 24.8% (14.4%) mean (SD) *p* = 0.1)	[54]
Swedish selegiline study	No	UPDRS	Selegiline vs. placebo	Positive, but after 8 weeks of washout no difference	[55]
DATATOP	No	UPDRS	Selegiline vs. tocopherol	positive after 9 months. Endpoint was need for L-dopa therapy	[56]
SINDEPAR	No	UPDRS	Selegiline plus bromocriptine plus L-dopa	Positive effect of selegiline	[57]

FIT, functional imaging technique; PD, Parkinson’s disease; UPDRS, Unified Parkinson’s Disease Rating Scale, MDS-UPDRS, Movement Disorders Society-Unified Parkinson’s Disease Rating Scale, exemplary studies selected by the authors listed according to the publication date.
